# Reaction-Induced Phase Separation and Morphology Evolution of Benzoxazine/Epoxy/Imidazole Ternary Blends

**DOI:** 10.3390/polym13172945

**Published:** 2021-08-31

**Authors:** Jun Yue, Honglei Wang, Qian Zhou, Pei Zhao

**Affiliations:** 1 School of Biomedical Engineering, Sun Yat-sen University, Guangzhou 510006, China; yuejun3@mail.sysu.edu.cn; 2 Key Laboratory for Polymeric Composite and Functional Materials of Ministry of Education, Guangdong Engineering Technology Research Center for High-Performance Organic and Polymer Photoelectric Functional Films, School of Chemistry, Sun Yat-sen University, Guangzhou 510275, China; 3 State Key Laboratory of Polymer Materials Engineering, College of Polymer Sciences and Engineering, Sichuan University, Chengdu 610065, China; qianzhou_scu@126.com

**Keywords:** benzoxazine/epoxy blends, reaction induced phase separation, phase morphology, morphology evolution

## Abstract

Introducing multiphase structures into benzoxazine (BOZ)/epoxy resins (ER) blends via reaction-induced phase separation has proved to be promising strategy for improving their toughness. However, due to the limited contrast between two phases, little information is known about the phase morphological evolutions, a fundamental but vital issue to rational design and preparation of blends with different phase morphologies in a controllable manner. Here we addressed this problem by amplifying the difference of polymerization activity (PA) between BOZ and ER by synthesizing a low reactive phenol-3,3-diethyl-4,4′-diaminodiphenyl methane based benzoxazine (MOEA-BOZ) monomer. Results indicated that the PA of ER was higher than that of BOZ. The use of less reactive MOEA-BOZs significantly enlarged their PA difference with ER, and thus increased the extent of phase separation and improved the phase contrast. Phase morphologies varied with the content of ER. As for the phase morphological evolution, a rapid phase separation could occur in the initial homogeneous blends with the polymerization of ER, and the phase morphology gradually evolved with the increase in ER conversion until the ER was used up. The polymerization of ER is not only the driving-force for the phase separation, but also the main factor influencing the phase morphologies.

## 1. Introduction

Due to the good processability, excellent thermal and mechanical properties, thermosetting resin (TS) has been widely used in the coating, electrical, and aerospace fields [1,2,3,4,5]. Unfortunately, TS resins are brittle materials that show insufficient toughness and elongation at break, which limits their use as structural materials. Effective toughening strategies are of high interest to enable new applications. Among the various toughening methods, reaction-induced phase separation, which was designed as the uniform precursor system undergoing a phase separation into a multiphase structure during the development of the curing process, was the most effective one [6,7]. This method could significantly improve the toughness of TS resin without sacrificing its high modulus, glass transition temperatures (*T*_g_), and excellent thermomechanical properties, which has been widely reported in high performance engineering thermoplastic (TP) (i.e., polyimide, poly(ether imide), and poly(ether sulphone)) modified TS systems [8,9,10,11,12]. However, the high viscosity of the resulted blends caused by the high molecular weight of TP resins limits their application in the advanced process technologies that require a resin matrix with low viscosity and good fluidity, such as the resin transfer molding (RTM) process [13,14]. Therefore, the development of advanced TS resin systems with low viscosity and high performance, especially excellent toughness, is still challenging and a new modification strategy is needed.

Using a low-viscosity and easily processible TS modifier to blend with TS matrix resins is one of the common methods used to obtain low-viscosity high-performance TS/TS matrix resins. Previously, numerous studies have been reported based on the structure–property investigation of TS/TS blending systems, viz., tuning parameters, including the composition of two resins, the types of catalyst, the structure of TS resins, and the curing procedures [15,16,17,18,19,20]. Although the overall performance of blends could be enhanced by rational control over these parameters, the toughness improvement was still limited. Inspired by the TP-modified TS systems, Gu’s group found that introducing multi-phase structures to TS/TS blending systems via reaction-induced phase separation (RIPS) could not only maintain the good processability of TS/TS blends but also significantly improve their toughness [21].

However, to achieve RIPS in TS/TS blending systems is not easy [22,23,24,25,26]. Firstly, unlike TS/TP blends, the two components in TS/TS systems are usually small molecular compounds, which means that the initial dynamical asymmetry (such as the differences in molecular weight, viscosity, and glass transition temperature) doesn’t exist. After the polymerization of one TS-component, the system still has a large entropy of mixing. Secondly, the two components can both polymerize to form a 3D crosslinked network. The poor initial dynamical asymmetry, huge entropy of mixing, as well as the inter-chain entanglement, the formation of interpenetrating polymer networks, and the co-polymerizations between two components are all unfavorable factors to the occurrence of phase separation. Recently, significant progress has been reported, focusing on strategies to achieve RIPS, the factors influencing the phase separation and the morphology–structure relationships in TS/TS blending systems. Li investigated the phase separation of benzoxazine (BOZ)/cyanate blends and obtained “sea-island” structures in situ by adjusting the chemical structure of BOZ resin and the molar ratio [27]. Wang prepared bi-continuous phase structures in BOZ/maleimide blends under the catalysis of imidazole (IMZ) and found that the viscosity (fluidity) of blends plays an important role in determining phase separation and phase morphology [28,29]. Zhang fabricated sea-island structures in BOZ/epoxy (ER) blends by varying the alkyl chain length of hyperbranched polymeric ionic liquids catalyst [30]. Previously, we investigated the RIPS behavior in BOZ/ER systems and the results showed that either increasing the initial molecular weight of ER or adding imidazole as the catalyst to realize sequential polymerization of ER and BOZ, are both beneficial for the phase separation. Through adjusting the composition ratio of the two components, we obtained a series of phase separation structures with different morphologies, such as sea-island structures, co-continuous structures, and inversed phase structures [21,31,32,33]. In addition, our studies indicated that the introduction of phase separation structures into BOZ/ER blends could improve the impact strength by 50% compared to the homogeneous system, while at the same time, the viscosity of the matrix resins, the high-modulus, and glass transition temperatures were well maintained. [34]

Although much progress has been made, comparing with the TS/TP systems, phase separation study for the TS/TS blending systems is still in its early stages and there are still many problems that remained to be overcome. For example, the phase separation extent in the current TS/TS blending system is still very low and the contrast between two phases is too ambiguous to be observed without an etching process. Without doubt, these challenges impede the investigation of the morphological evolutions, an important issue to understand the roles of each component in the phase separation and realize the preparation of TS/TS blends with different phase morphologies in a controllable manner. Therefore, developing a way to increase the phase separation extent in the blending systems to obtain an intuitional and clearer phase morphology is of great significance to promote the RISP studies in TS/TS blending systems.

Recently, we successfully fabricated multiphase structures in phenol-4,4′-diaminodiphenyl methane based benzoxazine resin (MDA-BOZ)/diglycidyl ether of bisphenol A epoxy resin (DGEBA-ER) under the catalysis of imidazole (IMZ) and systematically investigated the polymerization activity (PA) of each component and the effect of their PA differences on the phase separation. The results indicated that in MDA-BOZ/DGEBA-ER blends, the catalytic activity of IMZ to the DGEBA-ER is much higher than that to the MDA-BOZ, and the PA difference between DGEBA-ER and MDA-BOZ resin could be enlarged by increasing the content of IMZ; the larger the PA difference, the easier the phase separation; otherwise, homogeneous structures were obtained [34]. Furthermore, we found that impurities containing phenolic hydroxyl groups in the BOZ raw materials, which are hard to remove, could co-polymerize with ER and thus cause adverse effects on the phase separation. Here, we prepared a novel phenol-3,3-diethyl-4,4′-diaminodiphenyl methane based benzoxazine resin (MOEA-BOZ) with a high purity. Compared to the traditionally used MDA-BOZ, the polymerization activity of MOEA-BOZ significantly decreased. This property decreased the possible co-polymerizations and enlarged the PA difference between BOZ and ER, respectively, which finally increased the extent of phase separation and improved the phase contrast. We could therefore, for the first time, systematically monitor the phase morphological evolutions of BOZ/ER blends and dispense with the etching processes. In addition, we proposed a phase separation mechanism for the MOEA-BOZ/DGEBA-ER/IMZ blends based on the morphological evolutions and corresponding residual curing exothermic curves after curing at different stages obtained by differential scanning calorimetry (DSC) measurement. The current study provides a fundamental understanding of RIPS in TS/TS blending systems, which is expected to play a role in guiding the rational design of blends bearing phase separation structures for the purpose of toughness improvement.

## 2. Materials and Methods

### 2.1. Materials

**Scheme 1:**
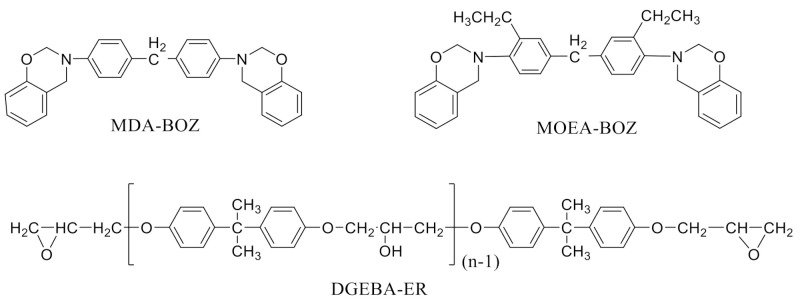
The chemical structures of MDA-BOZ, MOEA-BOZ and DGEBA-ER.

Phenol-4,4′-diaminodiphenyl methane based benzoxazine (MDA-BOZ) monomer with a melting temperature of 129 °C was synthesized, purified, and characterized following the previously reported method [35]. Diglycidyl ether of bisphenol-A epoxy resin (DGEBA-ER) (Shanghai Synthetic Resin Factory, Shanghai, China) with an epoxy equivalent of 227~246 g eq^−1^; Paraformaldehyde, Salicylaldehyde, Sodium borohydride (NaBH_4_), and imidazole (IMZ, *T*_m_ = 90 °C) (Chengdu Kelong Chemical Reagents Corp., Chengdu, China); and 3,3-diethyl-4,4′-diaminodiphenyl methane (MOEA) (Chang Shu Yongli Chemical Co., Ltd., Changshu, China) were all used as received without further purification. The chemical structures of MDA-BOZ and DGEBA-ER are shown in Scheme 1.

### 2.2. Synthesis of Benzoxazine

Phenol-3,3-diethyl-4,4′-diaminodiphenyl methane based benzoxazine resin (MOEA-BOZ) was synthesized via a three-step procedure starting from salicylaldehyde (Scheme S1), the method of which has been reported in many studies [36,37,38]. 4,4’-Methylene bis(2-ethylbenzenamine) (MOEA, 147.5 g, 0.5 mol) and salicylaldehyde (115.5 g, 1.1 mol) were dissolved into ethanol and stirred under N_2_ reflux for 4 h at 0~10 °C with a pH of 5~6. After filtration and drying, a brilliant-yellow powder was obtained (intermediate product A). Then, product A was redissolved in ethanol, and the pH was adjusted to 9 by the addition of NaOH solution. After cooling the solution to 0 °C with an ice bath, NaBH_4_ (56.7 g, 1.5 mol) was fed into the solution step by step and continuously stirred at 0 °C for 48 h under N_2_ reflux. After the reaction, the solution was precipitated into 1500 mL water and white powder (intermediate product B) was obtained after filtration and drying. Finally, a mixture of product B and paraformaldehyde (33 g, 1.1 mol) was dissolved in toluene and stirred at room temperature for 5 h. A bright red solid was obtained after removing the solvent under vacuum. The resulting solid was further purified by recrystallization in toluene/ethanol mixed solvent, and a white crystalline solid was obtained, and named as MOEA-BOZ. The chemical structure of MOEA-BOZ monomer was shown in Scheme 1.

### 2.3. Preparation of BOZ/DGEBA-ER/IMZ Blends

BOZ/DGEBA-ER blends were prepared by mixing BOZ (MDA-BOZ resin or MOEA-BOZ monomer, 18 g, 14 g, 10 g, or 6 g) and DGEBA-ER resin (2 g, 6 g, 10 g, or 14 g) with the mass ratios of 9/1, 7/3, 5/5, and 3/7 in a 100 mL eggplant-type flask at 100 °C for 20 min, and after the mixture had cooled to 95 °C, IMZ (12 wt% proportion to the total DGEBA-ER, 0.24 g, 0.72 g, 1.2 g, or 1.68 g, respectively) was added and the mixture was stirred vigorously for 2 min until IMZ was completely dissolved. The samples were degassed under vacuum for another few minutes afterwards and then refrigerated to avoid any further curing reaction. To investigate the morphology evolution, samples at different curing stages were prepared by casting the above blends into metal molds and curing with a profile as following: 110 °C/24 h, 130 °C/2 h, 150 °C/2 h, 180 °C/2 h, and 200 °C/2 h.

### 2.4. Measurements

To investigate the polymerization activity of BOZ, DGEBA-ER and their blends with IMZ, DSC experiments were performed on a TA Q20 instrument (TA Instruments, New Castle, DE, USA) under nitrogen flow of 50 mL/min. For measurements of MOEA-BOZ monomers, 3~5 mg of sample encapsulated in an aluminum pan was equilibrated at 0 °C and then heated to 120 °C (higher than the melting temperature (*T*_m_) but lower than the curing temperatures) at a rate of 10 °C/min. The endothermic peak temperature corresponds to the *T*_m_ of MOEA-BOZ monomers. Subsequently, the sample was cooled to 30 °C at a rate of 10 °C/min to study whether crystallization would occur during the cooling process. To determine the curing temperature of MOEA-BOZ, a third scan was performed from 30 °C to 350 °C at a rate of 10 °C/min. For other systems, only one scan was employed to investigate their polymerization activity, which was performed under a heating scan from 40 °C to 350 °C at a rate of 10 °C/min.

Phase morphology of each blend was investigated by Inspect-F field emission scanning electron microscope (SEM) (FEI, Hillsboro, OR, USA) (at an acceleration voltage of 20 KV. Samples for SEM measurement were fractured under liquid nitrogen. The fractured surfaces were totally dried at room temperate under vacuum and coated with gold before SEM observation. The composition of each rich-phase was investigated by Fourier transform-infrared spectroscopy (FTIR) or attenuated total reflectance (ATR)-FTIR measurement, which was performed on a Nicolet 5700 FTIR spectrometer (Thermo Fisher Scientific, Waltham, MA, USA) with an average of 32 scans at a resolution of 4 cm^−1^. Samples for FTIR were prepared by casting THF solutions onto a potassium bromide window at room temperature, while samples for ATR-FTIR were THF-insoluble gels (around 5 mm × 5 mm × 3 mm) which were totally dried under vacuum at room temperature overnight before measurement. Proton nuclear magnetic resonance (^1^H NMR) spectrum was recorded on a TD-65536 NMR (Bruker, Billerica, MA, USA) with the frequency of 400 MHz in DMSO-*d*_6_.

## 3. Results and Discussion

### 3.1. Synthesis of MOEA-BOZ Resin

In this study, we prepared the MOEA-BOZ monomer via a three-step procedure (Scheme S1). First, we synthesized an intermediate product A bearing a Schiff base structure by coupling 3,3-diethyl-4,4′-diaminodiphenyl methane (MOEA) with a twofold molar ratio of salicylaldehyde. The appearance of a C=N absorption peak at 1607 cm^−1^ (Figure S1a) in the FTIR spectra indicated the successful synthesis of compound A. Second, using sodium boron hydroxide, the imine bond in compound A was successfully reduced to a C–N bond (intermediate product B), which was verified by the disappearance of FTIR band at 1607 cm^−1^ and the appearance of a new band at 3372 cm^−1^ (Figure S1b) corresponding to the absorption peak of secondary amine. Finally, the MOEA-BOZ monomer was synthesized via the intramolecular cyclization reaction of intermediate product B in the presence of formaldehyde. The crude product could be further recrystallized from the acetone/ethanol mixed solvent to give a white crystal product with a high purity. As shown in Figure S1c, absorption peaks were observed at 940 cm^−1^ (oxazine ring); 1031 and 1228 cm^−1^ (C-O-C stretching vibration); 1112 cm^−1^ (C-N-C stretching vibration); 1338 cm^−1^ (CH_2_ of oxazine ring, breathing vibration); 881, 823, and 754 cm^−1^ (substituted phenyl groups); 2963 and 2929 cm^−1^ (C-H stretching vibration), indicating that the obtained white crystals were the target product MOEA-BOZ.

**Figure 1:**
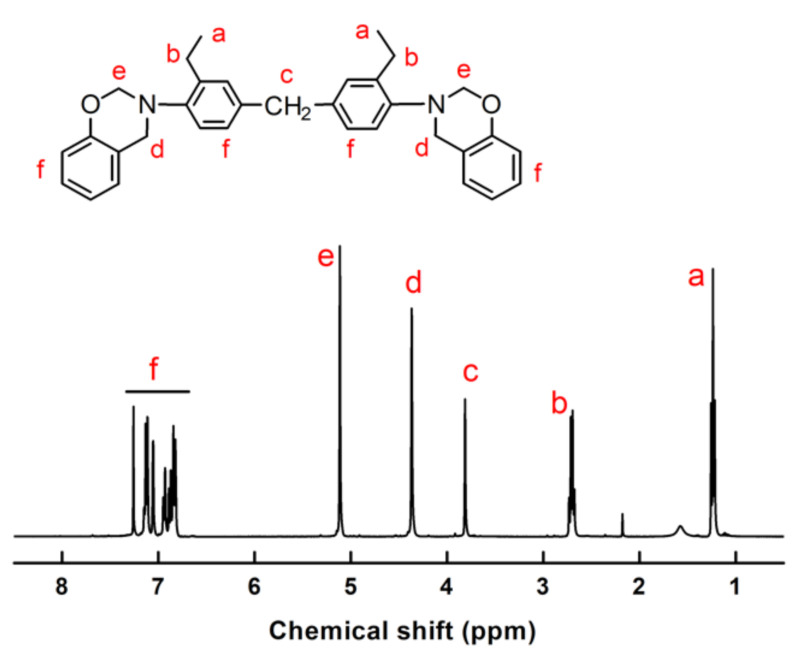
^1^HNMR spectrum of MOEA-BOZ monomer.

Furthermore, proton nuclear magnetic resonance (^1^H-NMR) experiments were carried out to characterize the chemical structure of MOEA-BOZ. As shown in Figure 1, resonances at 1.2 ppm (a), 2.7 ppm (b), and 3.7 ppm (c) came from the original -CH_3_, -CH_2_- (adjacent to the methyl group) and methylene bridge (between two phenyl groups) of reactant MOEA. Multiple resonances at 6.77–7.34 ppm correspond to the hydrogens on the phenyl rings (f). While resonances at 4.2 ppm (d) and 5.1 ppm (e) were attributed to methylene of N-CH_2_-Ar and N-CH_2_-O in the newly formed oxazine rings, respectively. The integral areas of the resonance peaks at 4.2 and 5.1 ppm were nearly the same, indicating the stoichiometric cyclization reaction of intermediate product B with formaldehyde. The as formed MOEA-BOZ monomer had a high purity (≈99%).

### 3.2. The Curing Reaction of MOEA-BOZ Monomer

**Figure 2:**
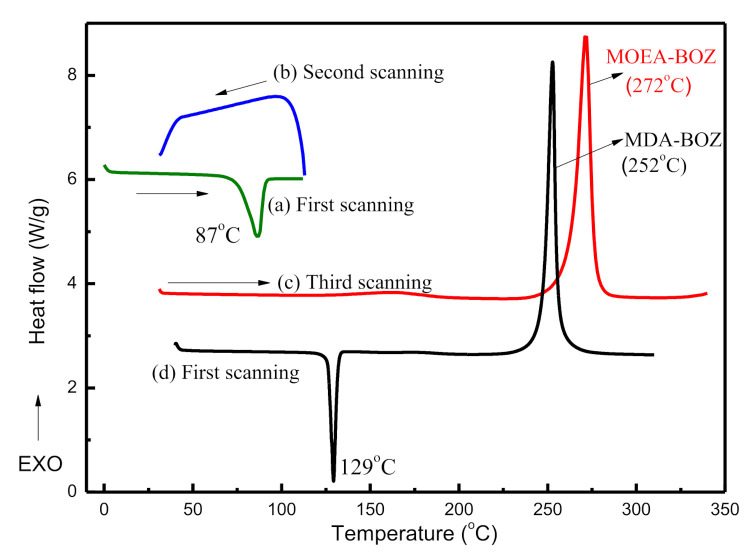
DSC curves of MOEA-BOZ (**a**–**c**) and MDA-BOZ (**d**) monomers.

The polymerizations of BOZ follows a cationic ring-opening polymerization mechanism [39]. The substitutes of aromatic rings next to the N atoms of oxazine ring don’t change their polymerization mechanisms, but will influence the polymerization activity (PA) of BOZ due to the electronic effects or stereo-hindrance effect [35]. To investigate the PA of BOZ, the differential scanning calorimeter (DSC) technique was employed. Because MOEA-BOZ is a new type of BOZ monomer, we have no information about its melting temperature (*T*_m_), crystallizability, or curing temperature. To determine these three parameters, we performed the heating–cooling–heating cycle with different temperature settings at each scan. The results are shown in Figure 2. From the first scanning cycle (Figure 2a), we found that MOEA-BOZ monomers had a melt temperature at 87 °C, much lower than that of MDA-BOZ monomers (129 °C, in Figure 2d), indicating that the introduction of ethyl groups on the phenyl ring decreased intermolecular interactions within crystals. Following the complete melting of MOEA-BOZ, a cooling scan from 120 °C to 30 °C was performed at a rate of 10 °C/min to investigate the possible crystallization behavior of MOEA-BOZ. However, no crystallization exothermic peak was observed during the whole cooling process (Figure 2b). During the third scan, where MOEA-BOZ resin was gradually heated from 30 °C to 350 °C at a heating rate of 10 °C/min, neither cold crystallization peak nor melting peak could be observed before 200 °C. While continuously heating samples to 350 °C, an exothermic peak at around 272 °C was observed (Figure 2c), which could be attributed to the curing reaction of MOEA-BOZ. In comparison with the exothermic curve of MDA-BOZ, we found that both the onset temperature and the peak temperature of MOEA-BOZ were 20 °C higher than those of MDA-BOZ, indicating that introduction of ethyl groups on the phenyl ring decreased the polymerization activity of MOEA-BOZ.

From the above analysis, we can see that compared to traditional MDA-BOZ resins, the reduction in structural symmetry of MOEA-BOZ led to a lower melting temperature as well as the crystallization capability, and decreased the polymerization activity. Therefore, MOEA-BOZ was expected to be ideal formulation to meet the requirements of our experimental designs: (1) high purity (to avoid the influence of phenolic hydroxyl groups on the curing reaction); (2) decreased crystallization capability (will not separate out from the homogenous phase and thus exclude the possibility of crystallization-induced phase separation within BOZ and ER blending systems); (3) lowered polymerization activity, which would be beneficial to increase the difference in polymerization activity between BOZ and ER.

### 3.3. Curing Reaction of MOEA-BOZ/DGEBA-ER/IMZ Blending Systems

**Figure 3:**
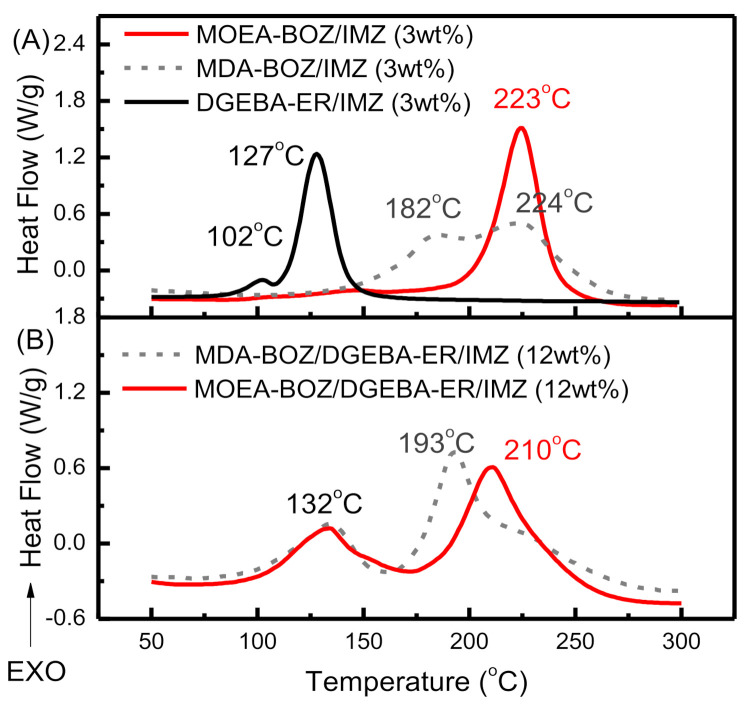
The DSC exothermal curves of MDA-BOZ, MOEA-BOZ, DGEBA-ER and their blends (7:3) with IMZ.

It has been proven in a previous paper that the polymerization of DGEBA-ER resin would not polymerize in the absence of a catalyst over the temperature range of 0~300 °C [33]. With the addition of 3 wt% of IMZ catalyst, two exothermic peaks at 102 °C and 127 °C could be observed during the heating scan of DGEBA-ER (Figure 3A). The shoulder peak at 102 °C was attributed to the addition reaction between DGEBA-ER and IMZ with a molar ratio of 1:2, while the exothermic peak at 127 °C corresponds to the crosslinking reaction of DGEBA-ER under the catalysis of oxonium ions produced by ring-opening polymerizations of ER [34]. As for MDA-BOZ resin, a broad exothermic peak starting from 150 °C (peak temperature located at 182 °C and 224 °C) appeared in the presence of 3 wt% of IMZ; While under the same amount of IMZ, a sharp exothermic peak of MOEA-BOZ monomers starting from 200 °C (peak temperature located at 223 °C) was observed. The shift in peak temperature to the high-temperature region confirmed that the polymerization activity of MOEA-BOZ was much lower than that of MDA-BOZ even under the catalysis of IMZ. With 12 wt% of IMZ, the MDA-BOZ/DGEBA-ER blend displayed two obvious curing exothermic peaks at 132 °C and 193 °C and the MOEA-BOZ/DGEBA-ER blend showed two curing exothermic peaks at 132 °C and 210 °C (Figure 3B). By comparing with the DSC exothermic curves of DGEBA-ER and BOZ resins, the exothermic peak at low temperatures (50~175 °C) could correspond to the polymerization of ER, including the addition reaction between ER and IMZ, and the crosslinking reaction of ER. While the exothermic peak at high temperatures (175~300 °C) could be attributed to the polymerizations of BOZ resins, probably including catalytic or thermal curing of BOZ, and co-polymerizations of BOZ with residual epoxy groups. Compared to MDA-BOZ/DGEBA-ER/IMZ blends with the same composition, it’s clear that the peak temperatures corresponding to the polymerization of DGEBA-ER resin almost overlap, while the peak temperature reflecting the polymerization of BOZ shifted to a higher temperature region in MOEA-BOZ/DGEBA-ER/IMZ blends. As a result, the PA differences between DGEBA-ER and BOZ resin was enlarged by the less-reactive MOEA-BOZ, which was beneficial to achieve sequential polymerizations, and obtain a clearer phase morphology in MOEA-BOZ/DGEBA-ER/IMZ blends.

### 3.4. Phase Morphology and Compositions of MOEA-BOZ/DGEBA-ER/IMZ Blends

**Figure 4:**
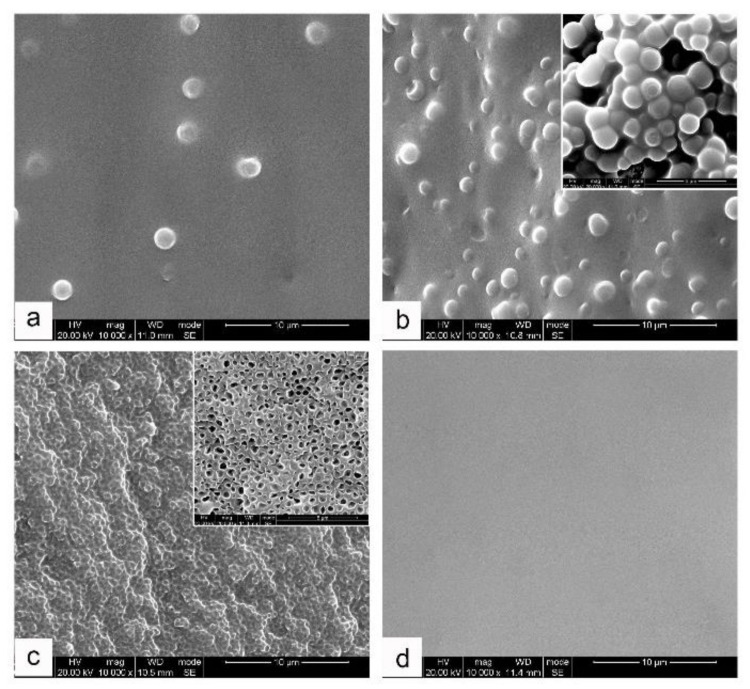
SEM morphology of MOEA-BOZ/DGEBA-ER/IMZ (12 wt%) blends with different content of ER after curing at 110 °C for 17 h: (**a**) 10 wt%, (**b**) 30 wt%, (**c**) 50 wt%, and (**d**) 70 wt%; the insert pictures were obtained after etching with THF for 5 min.

In previous SEM observations of MDA-BOZ/DGEBA-ER/IMZ blending systems, etching was essential to distinguish the two phases (Figure S2), which is unfavorable for studying the phase evolution process. However, for the current MOEA-BOZ/DGEBA-ER/IMZ systems, the phase structures could be clearly distinguished without etching, which enabled more accurate investigations of phase separation process. As shown in Figure 4, different morphologies were observed in MOEA-BOZ/DGEBA-ER/IMZ resins by varying the compositions of DGEBA-ER after curing at 110 °C for 17 h. When the composition of DGEBA-ER was not higher than 30 wt%, a sea-island structure was observed (Figure 4a), and with the increase in ER content, the population of dispersed ‘islands’ increased dramatically (Figure 4b). When the composition of ER reached to 50 wt% however, an inverse phase structure was observed (Figure 4c), where dispersed phases were trapped in the reticular continuous phase. Continuously increasing the ER content, no phase separation structures could be observed (Figure 4d).

Next, to investigate the chemical compositions of each phase for the above two typical blends bearing sea-island (Figure 4b) and inversed phase structures (Figure 4c), we separated the two phases by means of tetrahydrofuran (THF) etching utilizing the differences of polymerization degree for each rich phase after curing at 110 °C for 17 h, and analyzed their chemical information by IR measurement. For MOEA-BOZ/DGEBA-ER/IMZ with 30 wt% of DGEBA-ER, after etching with THF for 5 min, a large number of spherical particles with an average diameter of 1.5 μm were observed in SEM images of the THF-insoluble sections (Figure 4b, insert). When immersing the solid resins in THF for 24 h, the solid collapsed and a lot of flocculent precipitates appeared in the THF solution, which indicated that the flocculent precipitates corresponded to the dispersed phase in the blends with a high polymerization degree (gels), while the THF-soluble parts were attributed to the continuous phase in the blends with a low polymerization degree (solution). For MOEA-BOZ/DGEBA-ER/IMZ with 50 wt% of DGEBA-ER, after THF treatment for 5 min, a honeycomb-like structure with a wall-thickness around 300 nm was observed for THF-insoluble sections, corresponding to the continuous phase in the blends (Figure 4c, insert). When immersing the solid blends in THF for 24 h, the shape of solid did not change and the solution was still as clear as the pure THF, indicating that THF-insoluble sections attributed to the continuous phase had a high degree of polymerization (gel), while the 600~1000 nm holes in THF-dissolved parts were related to the dispersed phase (solution). Subsequently, we determined the chemical compositions of both THF-soluble and THF-insoluble parts by FTIR and ART-FTIR measurements, separately. As shown in Figure S3A,B, the FTIR spectra for THF-soluble part of the blends containing 30 wt% or 50 wt% of DGEBA-ER were nearly the same: where the characteristic bands for MOEA-BOZ monomers at 938 cm^−1^ (oxazine groups) and 756 cm^−1^ (1,2-substitued phenyl groups) were observed [40], indicating that the main composition of the THF-soluble part was unpolymerized MOEA-BOZ resin. Similarly, the FTIR spectra for the THF-insoluble parts from these two blends were also the same: the BOZ-relevant characteristic band at 943 cm^−1^ (oxazine groups) disappeared, and the ER-relevant characteristic bands at 1384 and 1362 cm^−1^ (isopropyl groups), 1039 and 1247 cm^−1^ (C-O-C groups), and 829 cm^−1^ (1,4-substitued phenyl groups) were observed, indicating that the main composition of the THF-insoluble part (the inserts of Figure S3A,B) was DGEBA-ER. The disappearance of characteristic bands at 911 cm^−1^ (epoxy groups), demonstrated that DGEBA-ER resins were completely polymerized.

From the above analysis, we can conclude that the dispersed spherical particles and the continuous matrix in Figure 4b corresponds to the ER-rich phase and the BOZ-rich phase, respectively. In contrast, the dispersed non-spherical particles and the continuous matrix in Figure 4c correspond to the BOZ-rich phase and ER-rich phase, respectively. The phase morphologies of MOEA-BOZ/DGEBA-ER/IMZ blends could be controlled through tuning the composition of DGEBA-ER: when the DGEBA-ER content was low enough (e.g., ≤30 wt%), sea-island structures where ER-rich phase dispersed in BOZ-rich matrix was observed; and both the size and the amount of dispersed ER-rich phases increased with the content of DGEBA-ER; When content of DGEBA-ER reached 50 wt%, an inversed phase structure where dispersed BOZ-rich phase was enveloped by reticulated ER continuous phase was obtained. When the DGEBA-ER content was high enough (e.g., 70 wt%), homogeneous structure was obtained.

### 3.5. Phase Morphology Evolution Process of MOEA-BOZ/DGEBA-ER/IMZ Blends

**Figure 5:**
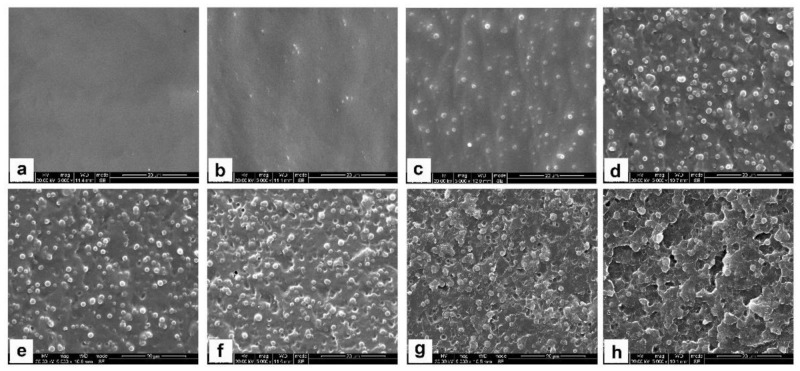
Morphological evolution process of MOEA-BOZ/DGEBA-ER/IMZ (12 wt%) blend with 30 wt% of ER at different curing stages: (**a**) before curing; after curing at (**b**) 110 °C/15 min; (**c**) 110 °C/65 min; (**d**) 110 °C/6 h; (**e**) 110 °C/11 h; (**f**) 110 °C/24 h + 130 °C/2 h + 150 °C/2 h; (**g**) 110 °C/24 h + 130 °C/2 h + 150 °C/2 h + 180 °C/2 h; (**h**) 110 °C/24 h + 130 °C/2 h + 150 °C/2 h + 180 °C/2 h + 200 °C/2 h.

To understand the morphological evolution of MOEA-BOZ/DGEBA-ER/IMZ blends with sea-island phase structures during the curing process, we prepared blends (containing 30 wt% of ER) at different curing stages, imaged their sections by SEM (Figure 5), and analyzed their phase domains via Image J software (National Institutes of Health, Bethesda, MD, USA) (Figure S4). As shown in Figure 5, after curing at 110 °C for 15 min, small number of spherical ER particles with a size of 0.5 μm, corresponding to the ER dispersed phase, could be observed (Figure 5b). With the process of curing reaction, more and more spherical ER particles appeared in the blends and their sizes gradually increased from 0.5 μm to 1.2 μm (Figure 5b–d). After curing at 110 °C for 6 h, the phase morphology as well as the particle size showed no significant changes either by extending the curing time (Figure 5e) or by elevating the temperature to 180 °C (Figure 5f,g). Continuously increasing the temperature to 200 °C, the boundary between two phases became blurred (Figure 5h).

**Figure 6:**
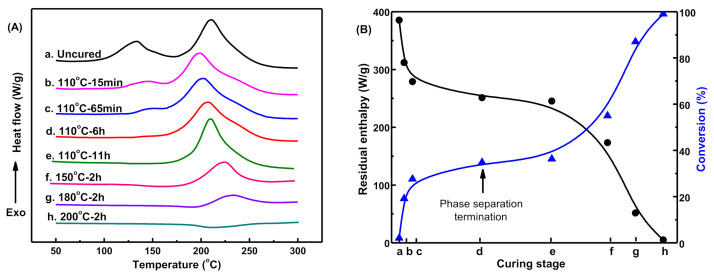
(**A**) DSC curves, (**B**) the Residual enthalpy and corresponding conversion of MOEA-BOZ/DGEBA-ER/IMZ (12 wt%) blend with 30 wt% of ER after curing at different stages.

To reveal the interconnections between the curing reaction and the morphological evaluations for blends in forming sea-island structures, we determined the exothermic behavior of blends at different curing stages by DSC. As shown in Figure 6A(a), the uncured MOEA-BOZ/DGEBA-ER/IMZ blend had two obvious exothermic peaks: the peak in the low-temperature region corresponds to the polymerizations of DGEBA-ER, while the peak in the high-temperature region corresponds to the curing of MOEA-BOZ. After curing at 110 °C for 15 min, the peak area corresponding to the polymerization of DGEBA-ER resin (*A*_DGEBA-ER_) declined rapidly and the system’s conversion reached 19.1% (Figure 6B(b)). At this stage, spherical ER particles were observed in Figure 5b, indicating that the occurrence of phase separation was induced by the polymerization of DGEBA-ER. With the process of curing reaction, the *A*_DGEBA-ER_ gradually decreased and disappeared after curing at 110 °C for 6 h, and the system’s conversion increased to 34.8% (Figure 6B(c,d)). During these periods, more and more spherical ER particles were observed and their sizes gradually increased (Figure 5c,d), which indicated that the morphology’s evolution was promoted by the increased conversion of DGEBA-ER. Afterwards, either extending the curing time or elevating the temperature, the peak area corresponding to polymerizations of MOEA-BOZ resin (*A*_MOEA-BOZ_) continuously decreased and the system’s conversion increased (Figure 6B(e–g)). While the phase morphology did not change any more (Figure 5e–g). After a further curing stage at 200 °C for 2 h, no exothermic peak was observed during the whole temperature scan, indicating the complete polymerization of two resins (Figure 6A(h)). The low contrast of the boundary between two phases observed in Figure 5h was probably due to the increased intermolecular crosslinking on the boundary (Figure 5h).

**Figure 7:**
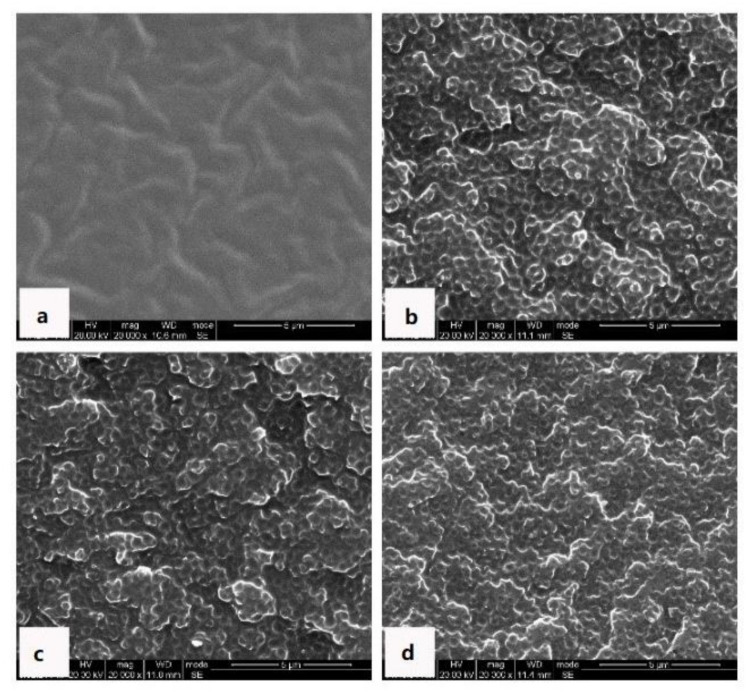
Morphological evolution process of MOEA-BOZ/DGEBA-ER/IMZ (12 wt%) blend with 50 wt% of ER after curing at different stages: (**a**) 110 °C/5 min; (**b**) 110 °C/40 min; (**c**) 110 °C/9 h; (**d**) 110 °C/24 h + 130 °C/2 h.

**Figure 8:**
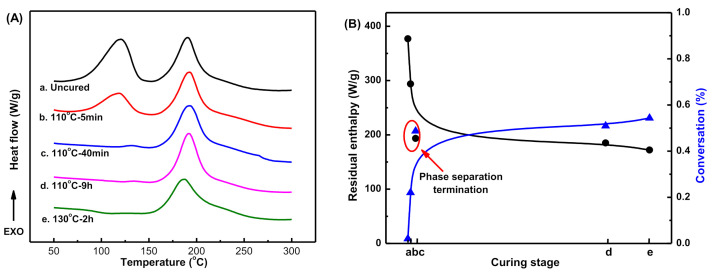
(**A**) DSC curves, (**B**) the Residual enthalpy and corresponding conversion of MOEA-BOZ/DGEBA-ER/IMZ (12 wt%) blend with 50 wt% of ER after curing at different stages.

Similarly, the morphological evolutions and the exothermic behavior of MOEA-BOZ/ER/IMZ blending systems with inversed phase structures bearing 50 wt% of ER were investigated by SEM and DSC measurements, respectively. As shown in Figure 7, phase separation phenomenon was observed after curing at 110 °C for just 5 min. The morphology at this stage was similar to the bi-continuous structure, although the contrast between the two phases was still too low (Figure 7a). After curing at 110 °C for 40 min, an inversed structure with the ER-rich phase as the continuous phase and BOZ-rich phase as the dispersed phase was observed (Figure 7b). After that, either extending the curing time or increasing the temperature did not change the phase morphology (Figure 7c,d). The exothermic behavior of blends at different curing stages is shown in Figure 8A. For the uncured resin mixture, two exothermic peaks appeared during the temperature scan (Figure 8A(a)). Due to the high content of DGEBA-ER (50 wt%), the value of *A*_DGEBA-ER_ was comparable with that of *A*_MOEA-BOZ_. After curing at 110 °C for 5 min, the *A*_DGEBA-ER_ had a rapid decline with the conversion of 22% (Figure 8A(b),B(b)). Combing the SEM images (Figure 7a) with the DSC analysis, it is clear that the fast polymerization of DGEBA-ER resin induced the phase separation at this stage. After curing at 110 °C for 40 min, the exothermic peak corresponding to the polymerization of the DGEBA-ER resin disappeared (Figure 8A(c)), and the conversion reached to 49.8% (Figure 8B(c)), along with the phase morphology change from the initial bi-continuous structure to an inversed phase structure (Figure 7b). Further extending the curing time or elevating the curing temperature would increase the conversion due to the polymerization of MOEA-BOZ resin (Figure 8B(d,e)), however, could not change the phase morphology anymore (Figure 7c,d).

### 3.6. The Plausible Mechanisms of Phase Separation in MOEA-BOZ/DGEBA-ER/IMZ Blends

From the above analysis of phase morphological evolutions and the related curing exothermic curves, we can see that the polymerization of ER promoted the phase separation, which was finished upon the exhaustion of ER monomers. Therefore, the phase separation mechanisms of MOEA-BOZ/DGEBA-ER/IMZ blends could be inferred from the relationship between the phase morphological evolutions and the polymerization of ER: when the phase separation occurred at 110 °C, only DGEBA-ER was polymerized while MOEA-BOZ barely reacted. (1) With the curing process, the molecular weight of ER gradually increased, which decreased the entropy of mixing and weakened its contribution to free energy of mixing, leading to the decrease in thermodynamic compatibility and thus was beneficial to the phase separation. (2) The polymerization of ER gradually increased the system’s viscosity and enlarged the dynamical asymmetry between ER polymers and MOEA-BOZ monomers. Due to the viscoelasticity of polymers, ER polymers tended to shrink to exclude the entrapped MOEA-BOZ molecules, leading to the phase separation as well as the phase morphology evolutions; (3) In addition to the role of promoting the phase separation, the polymerization of ER may cause the gelation of blends, which would adversely influence the phase separation. When the system contains a low content of ER (≤30 wt%), the polymerization rate of ER was relatively slow due to the dilution effect of MOEA-BOZ resins, which provides enough time for MOEA-BOZ to separate from the ER-rich phase and finally to form the sea-island structures. When the system contains a high content of ER, the polymerization rate of ER is very fast, which on the one hand could lead to a rapid phase separation, while on the other hand, could rapidly cause the gelation of the blends. In the latter situation, ER polymers would lose the viscoelasticity and thus would not be able to drive the separation of MOEA-BOZ from the ER polymer networks, leading to the formation of inverse phase structure or even homogenous structures. (4) At the high temperatures, although the polymerization of MOEA-BOZ would further decrease the entropy of mixing, ER polymers had lost their mobility due to the formation of crosslinked network structures and could not drive the phase separation. At the same time, the polymerization of MOEA-BOZ resins would cause the co-polymerizations with residual epoxy groups, which could also inhibit the phase separation.

Taken together, the polymerization of ER has two opposite influences on the phase separation, which are highly dependent on the compositions of ER and the curing stages. The polymerization of MOEA-BOZ at the later stage has nearly no influence on the phase morphological evolutions of blends. The trend of phase morphological evolutions is determined by the competition between the polymerization rate of ER and the speed of phase separation: increasing the polymerization rate of ER, e.g., via increasing the relative content of ER or IMZ, is beneficial to form an inversed or a homogenous phase structure. On the contrary, a sea-island phase structure may form.

## 4. Conclusions

In this study, we synthesized a novel MOEA-BOZ monomer with two ethyl groups modified on the phenyl rings of traditional MDA-BOZ resin. The introduction of ethyl groups decreased the molecular symmetry as well as the polymerization activity of MOEA-BOZ monomer, which enabled the sequential polymerization of ER and BOZ to achieve extensive phase separation in BOZ/ER/IMZ blends. The polymerization activity (PA) of BOZ or ER under the catalysis of imidazole (IMZ), the phase morphologies and their evolutions of BOZ/ER/IMZ blends were systematically investigated. Results showed that the PA of ER resin was higher than that of BOZ, and the use of less active MOEA-BOZ significantly enlarged the PA difference between ER and BOZ resins, therefore increased the extent of phase separation and improved the phase contrast. In addition, the phase morphology of MOEA-BOZ/ER/IMZ blends was highly dependent on the content of ER: when the ER content increased from 10 wt% to 50 wt%, the phase morphology of blends gradually changed from a sea-island structure with the ER-rich phase as the dispersed phase and the BOZ-rich phase as the continuous phase to an inversed phase structure with the ER-rich phase as the continuous phase and the BOZ-rich phase as the dispersed phase; Continuously increasing the content of ER, the blends displayed a homogenous structure with no phase separations. As for the phase morphological evolution, a rapid phase separation could occur in the initial homogeneous blends with the polymerization of ER, and the phase morphology gradually evolved with the increase in ER conversion until ER was used up. Further extending the curing time or enhancing the curing temperature increased the conversion of BOZ resin, while the phase morphology did not change. In general, the polymerization of ER in MOEA-BOZ/ER/IMZ blends, which on the one hand could result in decreased entropy of mixing, enhanced dynamical asymmetry, and increased polymer viscoelasticity, was the driving force of phase separation, while on the other hand could lead to the increase of system viscosity and chemical gelation, and thus to some extent was also harmful to the phase separation. The phase morphologies of MOEA-BOZ/DGEBA-ER/IMZ blends were the result of competition between the two opposing effects caused by the polymerization of ER.

## Data Availability

The data presented in this study are available on request from the corresponding authors.

## Supplementary Materials

The following are available online at https://www.mdpi.com/article/10.3390/polym13172945/s1, Scheme S1: Synthesis route of MOEA-BOZ monomers via a three-step procedure starting from salicylaldehyde; Figure S1: FTIR spectra of (a) intermediate product A, (b) intermediate product B, and (c) MOEA-BOZ; Figure S2: Phase morphology of MDA-BOZ blend with 10 wt% of DGEBA-ER and 8 wt% of imidazole after curing at 110 °C for 8 h. (left) without etching, (right) etched with THF for 5 min; Figure S3: FTIR spectra of MOEA-BOZ/DGEBA-ER/IMZ (12 wt%) blend with (A) 30 wt% and (B) 50 wt% of DGEBA-ER, the insets correspond to the phase morphologies of THF-insoluble parts.; Figure S4: The particle size distributions of MOEA-BOZ/DGEBA-ER/IMZ (12 wt%) blend with 30 wt% of DGEBA-ER after curing at different stages. The results were calculated via Image J software.
