# Supplementary material for: Reaction-Induced Phase Separation and Morphology Evolution of Benzoxazine/Epoxy/Imidazole Ternary Blends
Source: Polymers (Basel). 2021 Aug 31;13(17):2945. doi: 10.3390/polym13172945 (PMC8433963; doi:10.3390/polym13172945)
Supplement: Supplementary file 1 [file polymers-13-02945-s001.zip › polymers-1306945-supplementary.pdf]

Supplementary Materials

Reaction-induced Phase Separation and Morphology Evolution of Benzoxazine/Epoxy/Imidazole Ternary Blends

Jun Yue[1], Honglei Wang*[2], Qian Zhou[3], Pei Zhao*[2,3]

1. School of Biomedical Engineering, Sun Yat-Sen University, Guangzhou 510006, Guangdong, China
2. Key Laboratory for Polymeric Composite and Functional Materials of Ministry of Education, Guangdong Engineering Technology Research Center for High-performance Organic and Polymer Photoelectric Functional Films, School of Chemistry, Sun Yat-Sen University, Guangzhou 510275, China;
3. College of Polymer Sciences and Engineering, State Key Laboratory of Polymer Materials Engineering, Sichuan University, Chengdu 610065, China

* Corresponding author, Email: wanghlei3@mail.sysu.edu.cn, zhaopei55@mail.sysu.edu.cn

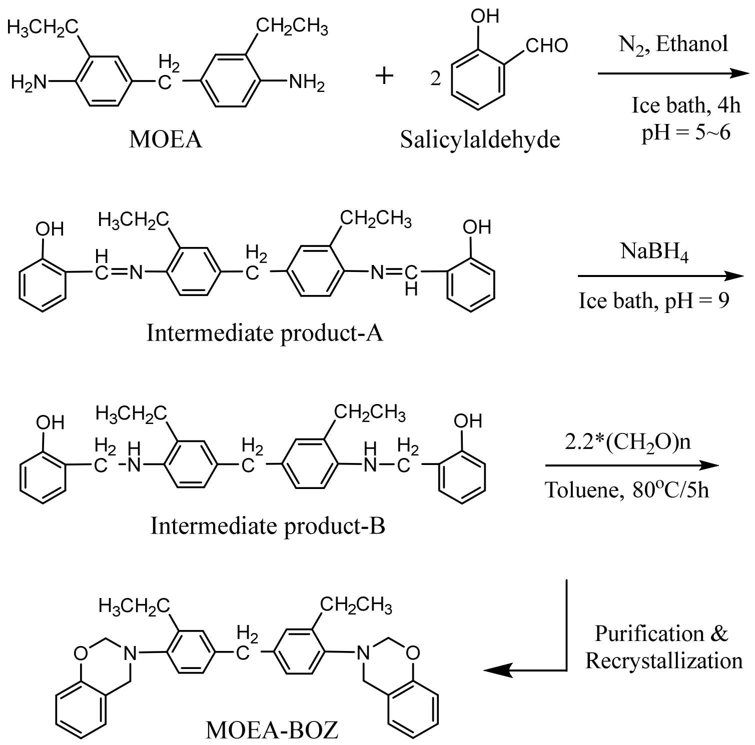

Scheme S1. Synthesis route of MOEA-BOZ monomers via a three-step procedure starting from salicylaldehyde.

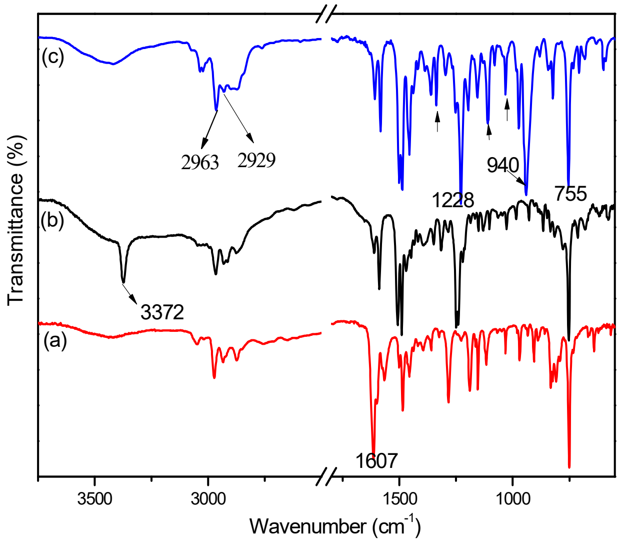

Figure S1. FTIR spectra of (a) intermediate product A, (b) intermediate product B and (c) MOEA-BOZ.

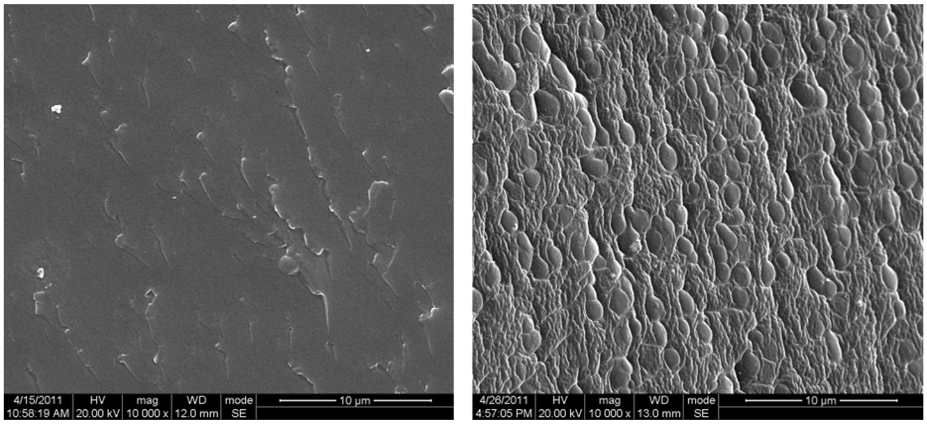

Figure S2. Phase morphology of MDA-BOZ blend with 10wt% of DGEBA-ER and 8wt% of imidazole after curing at 110 °C for 8h. (left) without etching, (right) etched with THF for 5min.

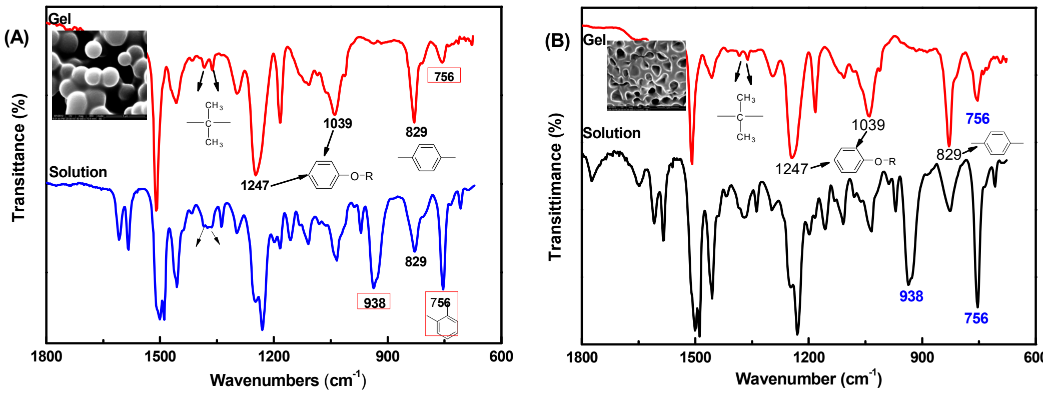

Figure S3. FTIR spectra of MOEA-BOZ/DGEBA-ER/IMZ (12wt%) blend with (A) 30wt% and (B) 50wt% of DGEBA-ER. The insets correspond to the phase morphologies of THF-insoluble parts.

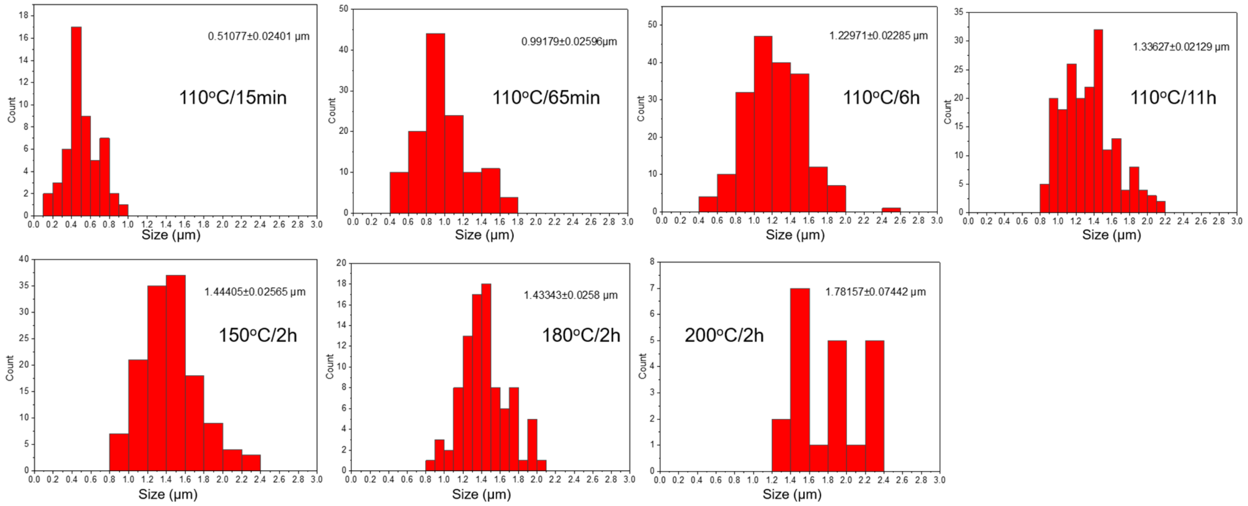

Figure S4. The particle size distributions of MOEA-BOZ/DGEBA-ER/IMZ (12wt%) blend with 30wt% of DGEBA-ER after curing at different stages. The results were calculated via Image J software (National Institutes of Health, Bethesda, MD, USA).
